# Magnetic and Electronic Evolutions of Hydrogenated VTe_2_ Monolayer under Tension

**DOI:** 10.1038/srep07524

**Published:** 2014-12-17

**Authors:** Hui Pan

**Affiliations:** 1 Institute of Applied Physics and Materials Engineering, Faculty of Science and Technology, University of Macau, Macao SAR, China

## Abstract

Two-dimensional nanostructures with controllable magnetic and electronic properties are desirable for their versatile applications in quantum devices. Here, we present a first-principles design on their magnetic and electronic switching controlled by tension. We find that hydrogenated VTe_2_ monolayer experiences a transfer from anti-ferromagnetism to ferromagnetism via a turning-point of paramagnetism, and switches from semiconductor, to metal, further to half-metal as tension increases. We show that its anti-ferromagnetism with semiconducting or metallic character under low tension is contributed to super-exchange or mobile-carrier enhanced super-exchange, while the ferromagnetism with half-metallic character under high tension is induced by carrier-mediated double exchange. We further show that the magnetic and electronic evolutions of hydrogenated VS_2_ and VSe_2_ monolayers under tension follow the same trend as those of hydrogenated VTe_2_ monolayer. We predict that tension is efficient and simple to control the magnetic and electronic properties of hydrogenated vanadium dichalcogenides monolayers. The monolayers with controllable magnetism and conductivity may find applications in multi-functional nanodevices.

Since the discovery of graphene^1^,^2^, two-dimensional (2D) nanostructures have attracted extensive attention because of their unusual physical, mechanical and chemical properties^1^,^2^,^3^,^4^,^5^,^6^,^7^,^8^. Among these 2D nanostructures, transition metal dichalcogenide (MX_2_) monolayers, where M is a transition metal element from group IV, group V, or group VI, and X is a chalcogen (S, Se or Te), have been widely investigated because of their easy fabrication, distinctive electronic, optical, and catalytic properties, and multi-functional applications^6^,^7^,^8^,^9^,^10^,^11^,^12^,^13^,^14^. Particularly, the physical and chemical properties of transition metal dichalcogenide monolayers can be easily tuned by controlling the composition, functionalizing, and applying external fields due to the abundant configurations of MX_2_, mechanic flexibility, and surface activity^15^,^16^,^17^,^18^,^19^,^20^,^21^,^22^. As one of important physical properties, the magnetism of these 2D monolayers has been a hot research area for their applications into quantum devices, such as spintronics. Ferromagnetism had been reported to be present in perfect VX_2_ (X = S, Se, and Te) monolayer^23^,^24^,^25^,^26^, NbS_2_ monolayers^16^, and zigzag MoS_2_ nanoribbons^5^,^15^. It was also reported that the ferromagnetism of 2D MX_2_ nanostructures can be efficiently enhanced by applying strain^15^,^16^,^17^ and controlled by hydrogenation^26^. For example, the magnetic properties of hydrogenated MoS_2_ monolayer can be tuned from non-magnetism, to ferromagnetism, and further to non-magnetism with the increase of tension^17^. The switching between the antiferromagnetic and ferromagnetic ground states, however, has not been realized in two-dimensional nanostructures. Here, we report the realization of the switching on hydrogenated vanadium telluride (VTe_2_-H) monolayer by applying tension on the basis of first-principles calculations. We find that VTe_2_-H monolayer can be tuned from antiferromagnetic state to paramagnetic state, and further to ferromagnetic state as tension increases. Accompanying with the magnetic evolution, we also show that its electrical character switches from semiconductor, to metal, and further to half-metal with tension increasing.

## Results and Discussion

**Figure 1:**
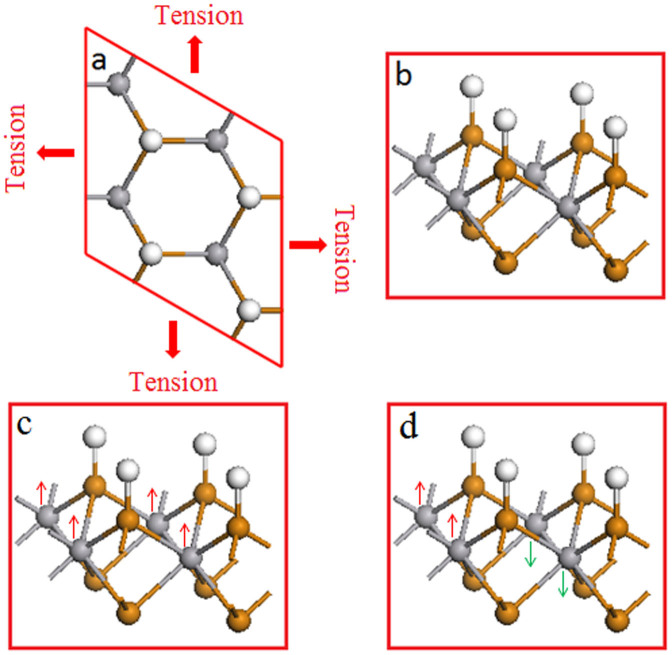
Representative structures of hydrogenated vanadium telluride (VTe_2_-H) monolayer. (a) top view with uniform static tension, (b) tilted view, (c) ferromagnetic spin-alignment, and (d) antiferromagnetic spin alignment.

The VTe_2_ monolayer with trigonal prismatic (2H) coordination and hydrogenation at one side (Figure 1) is first optimized to obtain the lattice parameters. A supercell with 4 unit cells (2 × 2 × 1) (Figure 1) is used to study its magnetic property. The ferromagnetic and anti-ferromagnetic spin alignments are shown in Figures 1c and 1d, respectively. The original lattice (c_0_ = 2a) is referring to its supercell's lattice under zero strain (7.462 Å). VTe_2_-H monolayer with extended lattice (c) is realized by stretching (see Figure 1a). The tension is defined as 
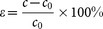
. A tension range of 0 to 15% is used in our calculations. The optimized structure shows that Te-V bond (Te without hydrogen cover) extends from 2.746 to 2.931 Å as the tension increasing from 0 to 15%, Te-V bond (Te with hydrogen cover) is 2.639 Å at zero tension and increases by 0.016 Å/1% tension, and Te-H bond is 1.721 Å at zero tension and increases by 0.001 Å/1% tension.

**Figure 2:**
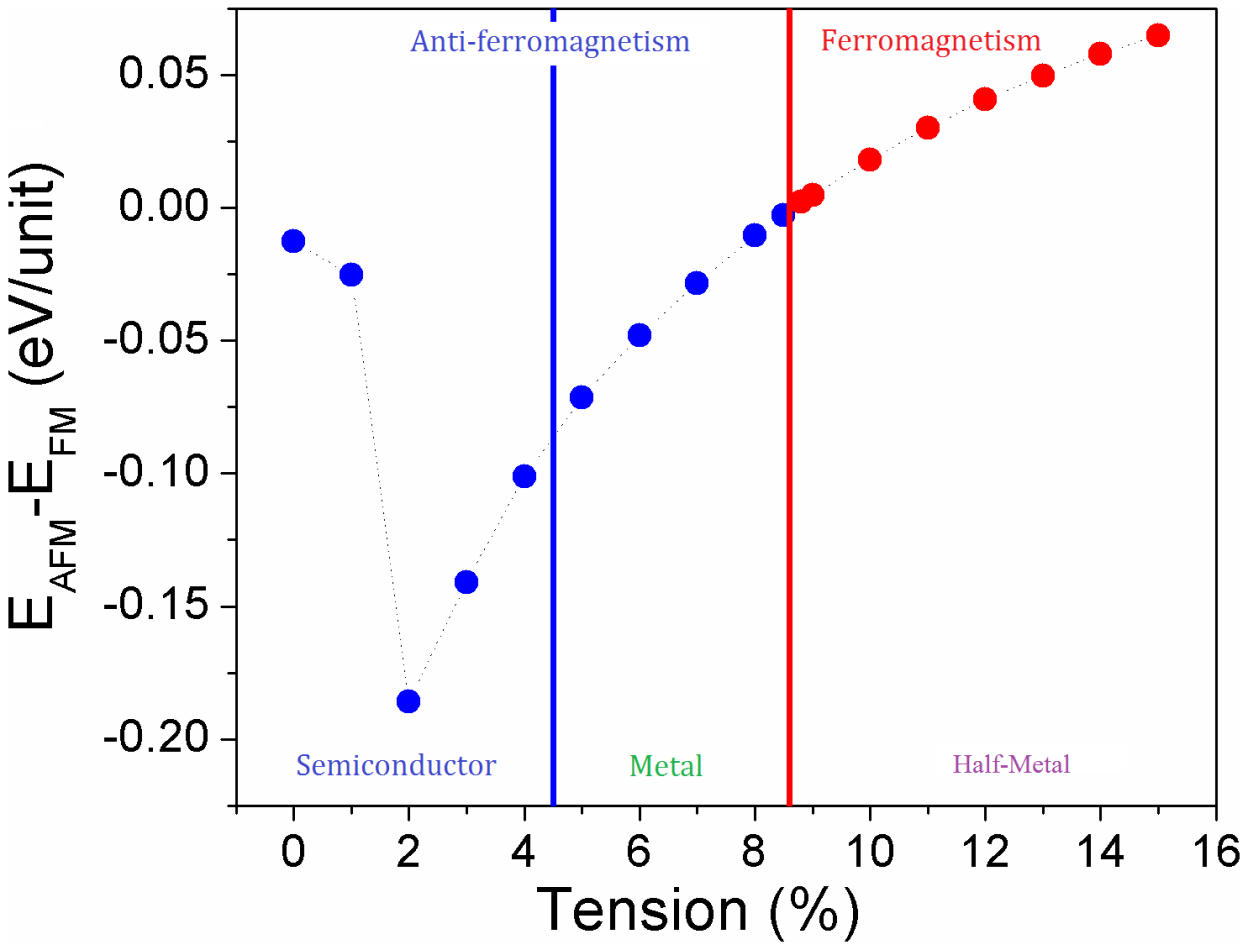
The calculated exchange energy of VTe_2_-H monolayer as function of tension. The regions with the evolution of magnetism and conductivity as tension are highlighted.

**Figure 3:**
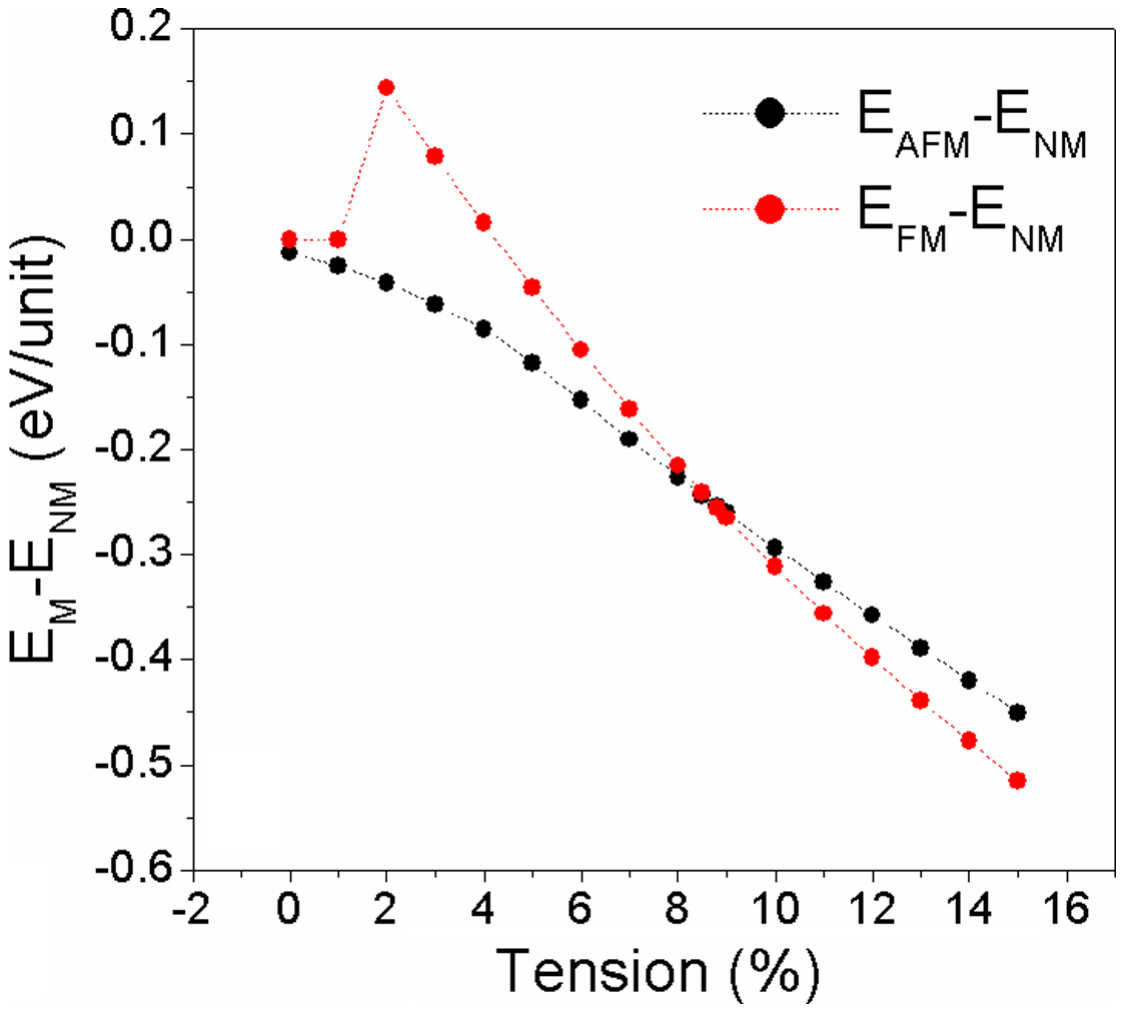
The calculated energy differences of VTe_2_-H monolayer between magnetic (antiferromagnetic/ferromagnetic) and non-magnetic states as function of tension.

To find out the magnetic evolution of VTe_2_-H monolayer under tension, the exchange energy (Δ*E_AFM-FM_*), defined as Δ*E_AFM_*–*_FM_* = (*E_AFM_* – *E_FM_*)/*N* (where *E_FM_* and *E_AFM_* are the energies at ferromagnetic and anti-ferromagnetic states, and N ( = 4) is the number of units in the supercell.), is calculated. We see that VTe_2_-H monolayer is anti-ferromagnetic with *E_ex_* = −13 meV under zero tension (Figure 2). The exchange energy (negative) initially decreases with the tension increasing and goes sharply down to bottom peak (−186 meV) at a tension of 2%. Then, the exchange energy (negative) increases with the tension increasing and reaches zero at a tension of ~8.6%, indicating that VTe_2_-H monolayer is anti-ferromagnetic with the tension less than 8.6%. Further increasing tension, the exchange energy becomes positive and increases with the applied tension, indicating that VTe_2_-H monolayer is ferromagnetic with the tension larger than 8.6%. To confirm the ground and metastable states of VTe_2_-H monolayer under tension, the energy difference between magnetic states (*E_FM_* and *E_AFM_*) and non-magnetic state (*E_NM_*), including ΔE_FM-NM_ ( = (*E_FM_* – *E_NM_*)/N) and ΔE_AFM-NM_ ( = (*E_AFM_* – *E_NM_*)/N), are calculated. The calculated energy difference between anti-ferromagnetic and non-magnetic states of VTe_2_-H monolayer shows that the anti-ferromagnetic state is more stable than non-magnetic state in the whole range of considered tension because its energy at anti-ferromagnetic state is lower than that at non-magnetic state (Figure 3). However, its ferromagnetic state is equivalent to its non-magnetic state when tension is less than 2%. At the tension of 2%, its ferromagnetic state becomes unstable as indicated by the larger positive ΔE_FM-NM_. Then, ΔE_FM-NM_ decreases with tension increasing and is negative after ε > 4%, indicating that ferromagnetic state is more stable than non-magnetic state when ε > 4%. We see that the ground state of VTe_2_-H monolayer is anti-ferromagnetic and its metastable state is no-magnetic at the tension range of 0 < ε < 5%, because ΔE_AFM-NM_ < 0 and ΔE_FM-NM_ ≥ 0. At the tension range of 4% < ε < 8.5%, both ΔE_AFM-FM_ and ΔE_FM-NM_ are negative (Figure 3), indicating that anti-ferromagnetic and ferromagnetic states of VTe_2_-H monolayer are ground and metastable states, respectively. At ε ~ 8.6%, ΔE_AFM-FM_ is equal to zero, but both of the energies of anti-ferromagnetic and ferromagnetic states are lower than that of non-magnetic state. So, we predict that VTe_2_-H monolayer is paramagnetic at ε ~ 8.6%. With further increasing tension, the energy of its ferromagnetic state is lower than that of anti-ferromagnetic states (positive ΔE_AFM-FM_ in Figure 2), and ΔE_AFM-NM_ keeps negative (Figure 3), confirming that the ground state of VTe_2_-H monolayer is ferromagnetic when ε > 8.6%, as well as its metastable anti-ferromagnetism. The calculated exchange energy and energy differences clearly show that the VTe_2_-H monolayer switches from anti-ferromagnetic state to ferromagnetic state via a paramagnetic state with the increment of applied tension (Figures 2&3). At its ferromagnetic ground state, the exchange energy (ΔE_AFM-FM_) can be up to 65 meV at a tension of 15%. On the basis of mean field theory and Heisenberg model with long-range interaction, the Curie temperature (*T_C_*) can be estimated from 
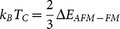
27. We see that the Curie temperature increases with the increase of tension (ε > 8.6%) because of the increased exchange energy (Figure 2). The estimated Curie temperature is 500 K for VTe_2_-H monolayer at a tension of 15%, indicating that they can be used in spintroincs at high temperature. The theoretically estimated Curie temperature should be confirmed experimentally.

**Figure 4:**
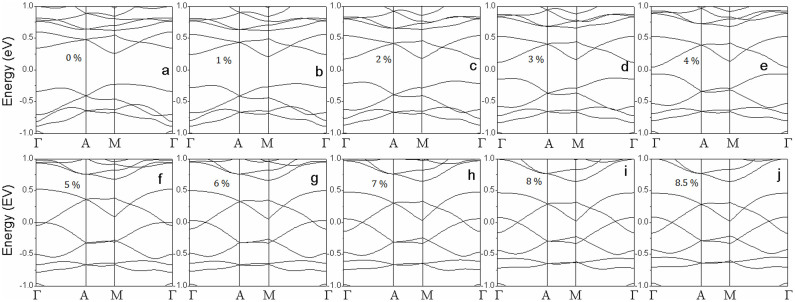
The calculated band structures of VTe_2_-H monolayers with anti-ferromagnetic ground states at a tension of 0% (a), 1% (b), 2% (c), 3% (d), 4% (e), 5% (f), 6% (g), 7% (h), 8% (i), and 8.5% (j).

**Figure 5:**
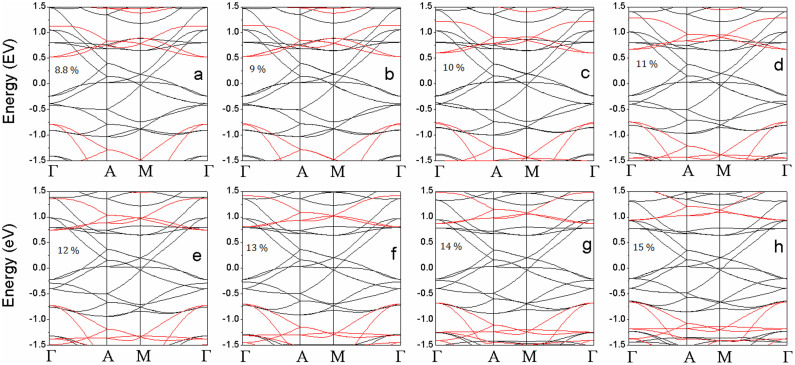
The calculated band structures of VTe_2_-H monolayers with ferromagnetic ground states under a tension of 8.8% (a), 9% (b), 10% (c), 11% (d), 12% (e), 13% (f), 14% (g), and 15% (h). Black line: spin-up; red line: spin-down.

To reveal the origin of the magnetic evolution with applied tension, the electronic structure of VTe_2_-H monolayer under tension is calculated. The calculated band structures show that VTe_2_-H monolayer under a tension up to 4% is a semiconductor (Figures 4a–e). The VTe_2_-H monolayer at zero tension is a direct band semiconductor with both of conduction band bottom (CBB) and valence band top (VBT) at M point and a gap of 0.48 eV (Figure 4a). As the tension increases, the conduction band and valence band at Γ point are pulled down and pushed up, respectively, and gradually become the CBB and VBT (Figures 4b–e), leading to the narrowing of band gap. At the tension of 4%, the direct band gap at Γ points is only 0.13 eV (Figure 4e). With further increasing the tension (>4%), CBB continuously moves down, and VBT up reversely (Figures 4f–j). At a tension of 5%, CBB and VBT cross each other at the Fermi level (0 eV in Figure 4), leading to the metallic conductivity of VTe_2_-H monolayer (Figure 4f). Its conductivity is further improved with the tension increasing up to 8.5%, because the overlap between conduction and valence bands increases in the same trend (Figures 4f–j). We can see that the conductivity of anti-ferromagnetic ground state of VTe_2_-H monolayer is continuously improved (from narrow-band semiconductor, to ultra-narrow-band semiconductor, further to metal) with the tension increasing from 0 to 8.5% (Figures 2&4). However, the conductivity of VTe_2_-H monolayer is changed as its magnetic ground state switches from anti-ferromagnetism to ferromagnetism under a tension above 8.6%. The calculated band structures of VTe_2_-H monolayers under a tension ranging from 8.8 to 15% clearly show that spin-up states are metallic, while spin-down states are semiconducting, resulting in half-metallic and ferromagnetic ground states (Figure 5). The change of conductivity (or carrier density) may provide evidence to find the mechanism on the magnetic evolution of VTe_2_-H monolayer with tension.

**Figure 6:**
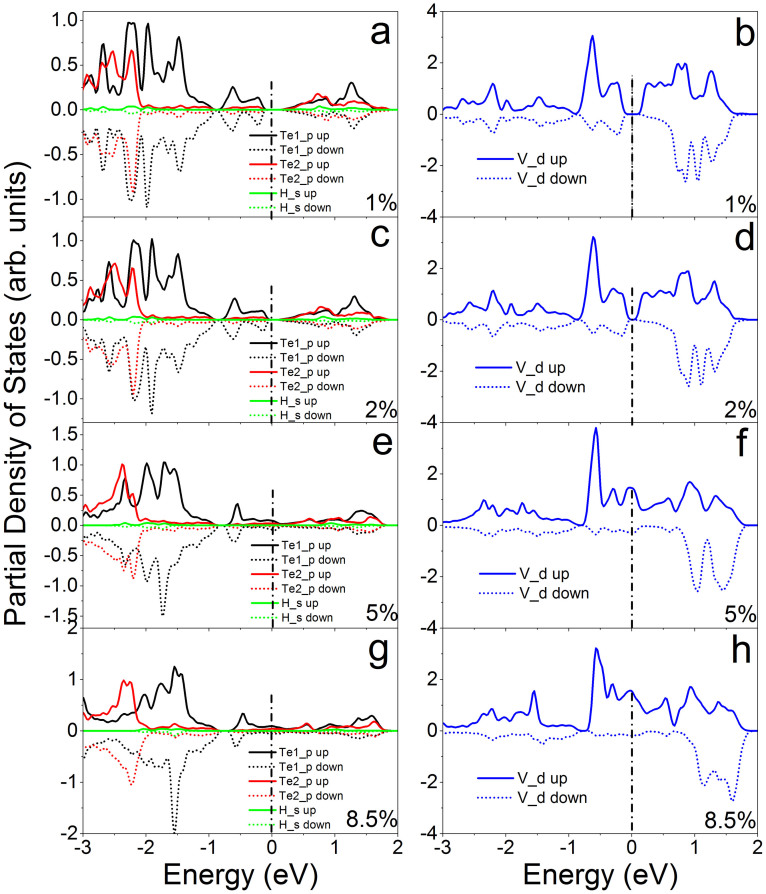
The calculated partial densities of states of VTe_2_-H monolayers with anti-ferromagnetic ground states at a tension of 1% (a) and (b), 2% (c) and (d), 5% (e) and (f), and 8.5% (g) and (h). Te1 and Te2 are Te atoms without and with H attachment, respectively.

**Figure 7:**
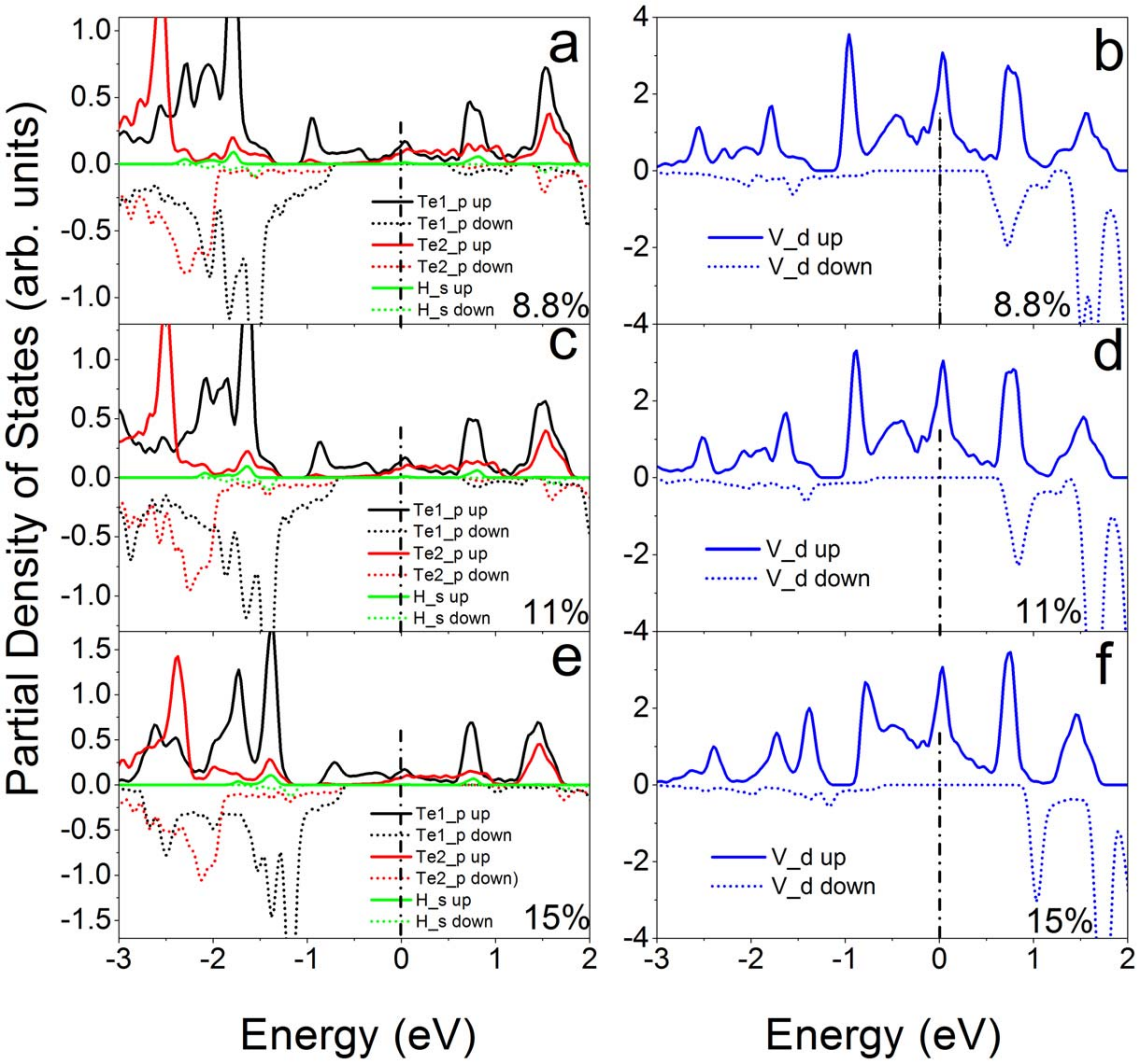
The calculated partial densities of states of VTe_2_-H monolayers with ferromagnetic ground states at a tension of 8.8% (a) and (b), 11% (c) and (d), and 15% (e) and (f). Te1 and Te2 are Te atoms without and with H attachment, respectively.

**Figure 8:**
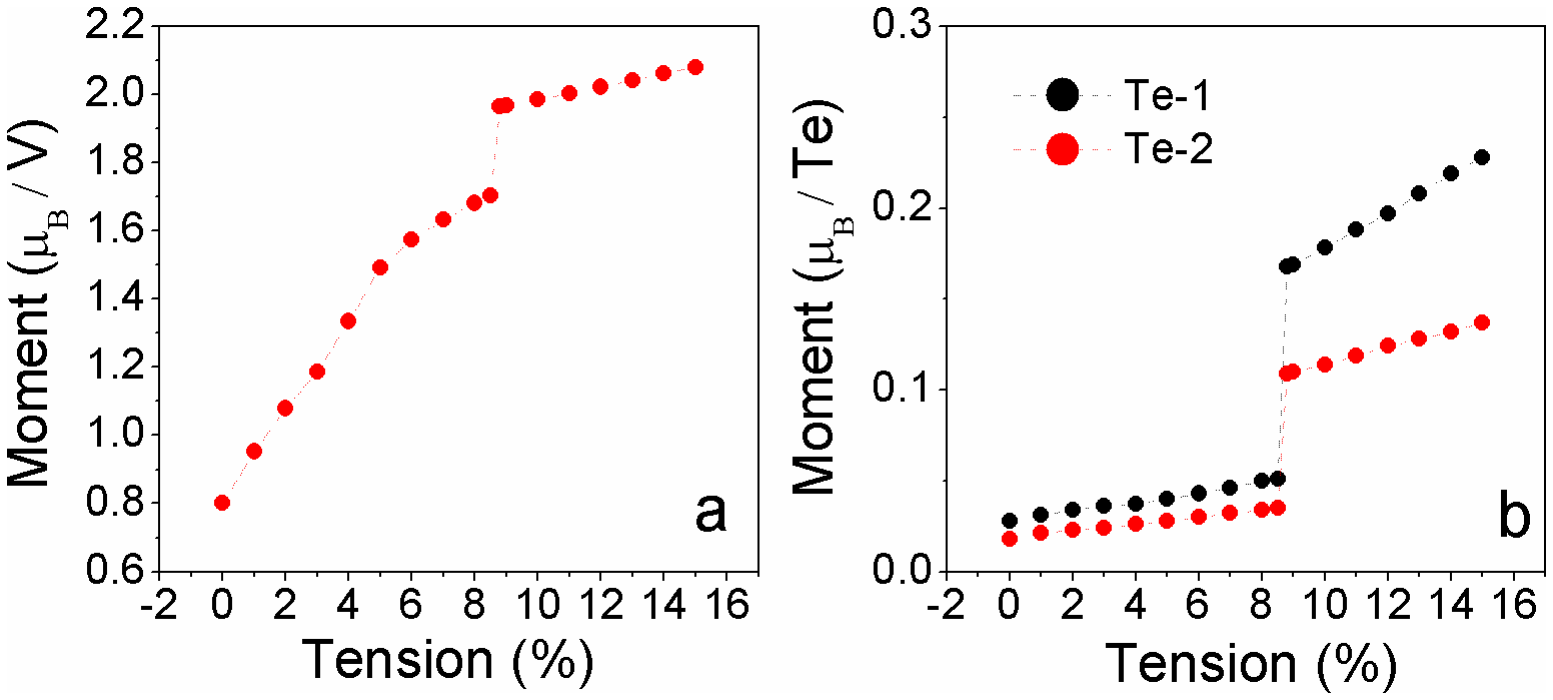
The calculated magnetic moments of VTe_2_-H monolayers as a function of tension. (a) V atom and (b) Te atoms (Te-1 andTe-2 are Te atoms without and with H attachment, respectively.).

**Figure 9:**
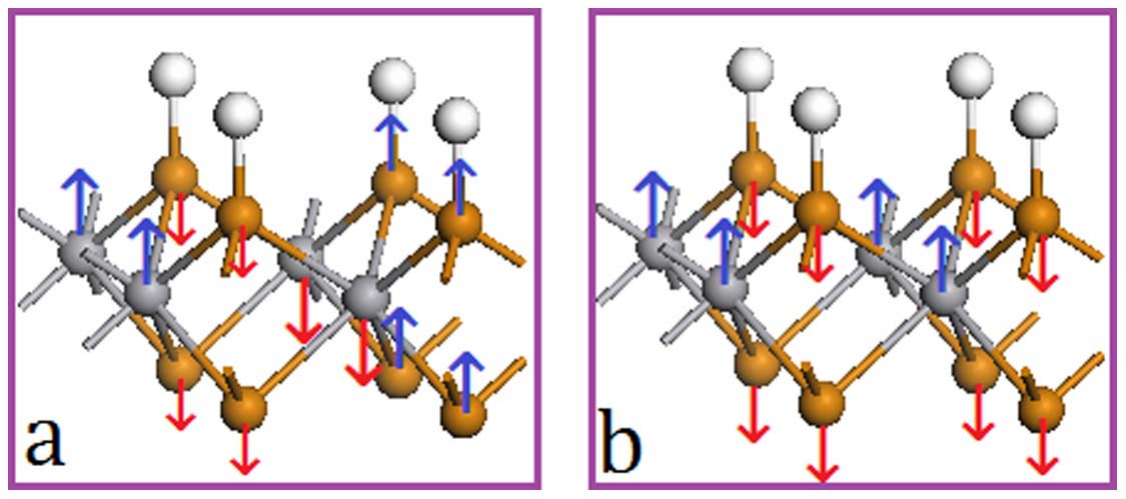
Alignment configurations of magnetic moments. (a) antiferromagnetic ground state and (b) ferromagnetic ground state.

Further analysis on the partial density of states (PDOSs) shows that the coupling between *d* electrons of V atoms (V_d) and *p* electrons of Te atoms (Te_d) of VTe_2_-H monolayer and their contribution to spin-polarized states and carrier near the Fermi level change as tension increasing from 0 to 15% (Figures 6&7), because of the charge redistribution. The PDOSs show that the hybridization between Te_p and V_d orbitals in semiconducting VTe_2_-H monolayer is strong under weak strain (Figures 6a~ d ), where these orbitals strongly couple below the Fermi level in a range of −0.8 to 0 eV and the covalent bonding is dominant. Under medium strain, the covalent-coupling between Te_p and V_d orbitals weakens, especially near the Fermi level (Figures 6e ~ h), leading to equivalent ionic and covalent bonding. Further increasing strain, the hybridization is weakened and ionic bonding becomes dominant (Figure 7). The H_s orbital hybridizes with Te_p orbitals around −2.0 eV below the Fermi level at a strain of 1%, which increases to around −1.3 eV with the strain increasing up to 15% (Figures 6&7). The calculated magnetic moments of V atom and Te atom are 0.79 and 0.02 μ_B_ at zero tension, respectively, and increase with tension, confirming the redistribution of charge (Figure 8). The moments of V atoms of VTe_2_-H monolayer under a tension ranging from 0 to 4% are anti-parallel because of its anti-ferromagnetic ground states (Figure 9a). Importantly, we see that the moments of Te atoms are also anti-parallel among neighboring cells (Figure 9a). From the anti-parallel alignment of magnetic moments between V and Te atoms and its semiconducting character, we see that super-exchange among V atoms, the mechanism of anti-ferromagnetic oxide insulators^26^,^28^,^29^, is achieved via Te atoms, and plays a dominant role on the anti-ferromagnetism of VTe_2_-1H monolayer in tension range of 0 to 4%. The alignments of magnetic moments keep unchanged in metallic VTe_2_-H monolayer in tension range of 5 to 8.5% (Figure 9a). The metallic anti-ferromagnetic ground state is also attributed to super-exchange, because the mobile carriers with high density can enhance the super-exchange interaction of anti-ferromagnetic metals^30^. Increasing tension from 8.6 to 15%, the VTe_2_-1H monolayer is half-metallic and ferromagnetic (Figure 5). We see that the magnetic moments of V and Te atoms jump up at the turning-point with a tension of ~8.8% (Figure 8). The magnetic moments of V and Te atoms are contributed to d and p electrons, respectively (Figure 7), and increase as tension (Figure 8). The magnetic moment of V atom can be up to 2.08 μ_B_ at a tension of 15%. The magnetic moments of Te atoms without and with H functionalization at a tension of 15% are 0.23 and 0.14 μ_B_, respectively. The PDOSs analysis shows that the moments of V atoms of VTe_2_-H monolayer at ε > 8.5% are parallel to each other because of its ferromagnetic ground states, and those of Te atoms are also parallel (Figure 9b). However, the moment alignment between V and Te atoms is anti-parallel (Figure 9b). The anti-parallel alignment between the magnetic moments of V and Te atoms and the half-metallic character of VTe_2_-H monolayer at ε > 8.6% demonstrate that double exchange is the dominant mechanism for the ferromagnetism^31^,^32^,^33^,^34^,^35^, where the exchange interaction is realized by the hopping of mobile carriers. That is, given the incomplete filling of bands (only spin-down bands are filled) (Figure 5), the band energy of the ferromagnetic state is lower than that of the antiferromagnetic state if a sufficient (usually rather small) number of carriers exist^36^. We see that the band gap of spin-up band structure of ferromagnetic VTe_2_-H monolayer increases with the increment of tension, resulting in the enhancement of spin-polarized electrons and larger magnetic moments due to the extended ionic bond strength of V-Te, which further confirms the dominant role of double-exchange for their ferromagnetism.

From the band structures, PDOSs, and spin-alignment of VTe_2_-H monolayer under tension, we see that magnetic and conducting properties can be controlled by external strain, and the magnetic switching is contributed to the change of its conducting character and the hybridization between Te_p and V_d orbitals under tension. According to the conducting character of VTe_2_-H monolayer under tension, there are three regions: semiconductor (ε ≤ 4%), metal (4% < ε < 8.6%), and half-metal (ε > 8.6%) (Figure 2). According to the magnetic property of VTe_2_-H monolayer under tension, there are two regions: anti-ferromagnetism (ε ≤ 8.5%) and ferromagnetism (ε > 8.6%) (Figure 2). Under low tension, the semiconducting or metallic VTe_2_-H monolayer is anti-ferromagnetic due to the super-exchange interaction or carrier-enhanced super-exchange. At high tension, the half-metallic VTe_2_-H monolayer is ferromagnetic due to carrier-mediated double exchange.

**Figure 10:**
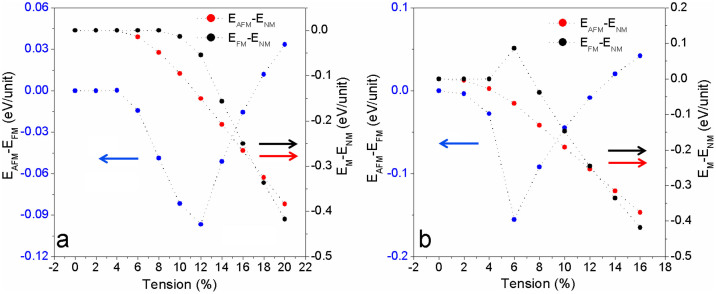
The calculated exchange energies and energy differences between magnetic (antiferromagnetic/ferromagnetic) and non-magnetic states as function of tension. (a) VS_2_-H and (b) VSe_2_-H monolayers.

**Figure 11:**
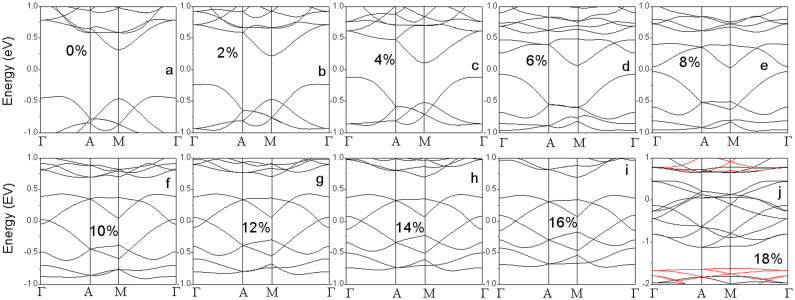
The calculated band structures of VS_2_-H monolayers under tension ranging from 0 to 18%. In (j), black line: spin-up; red line: spin-down.

**Figure 12:**
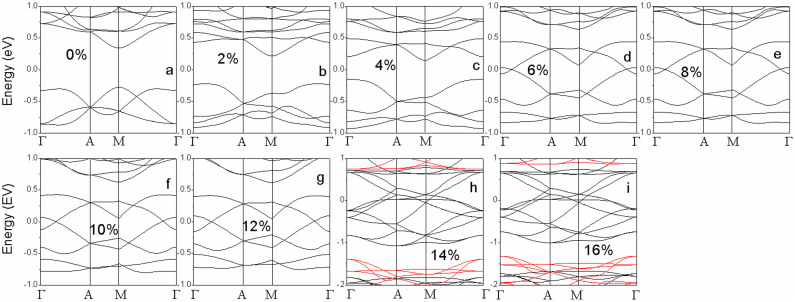
The calculated band structures of VSe_2_-H monolayers under tension ranging from 0 to 16%. In (h) and (i), black line: spin-up; red line: spin-down.

To further confirm the origin of magnetic evolution of VTe_2_-H monolayer under tension, we investigate the electronic and magnetic properties of hydrogenated vanadium disulfide (VS_2_-H) and diselenide (VSe_2_-H) monolayers under tension. Similar to VTe_2_-H monolayer, the calculated exchange energies demonstrate that VS_2_-H and VSe_2_-H monolayers switch from anti-ferromagnetism to ferromagnetism via paramagnetic turning points as tension increases (Figure 10). Differently, VS_2_-H and VSe_2_-H monolayers are non-magnetic at tension ranges of ε ≤ 4% and ε ≤ 2%, respectively, because the exchange energies and energy differences between magnetic and non-magnetic states are zero (Figure 10). The VS_2_-H monolayer is an intrinsic semiconductor with a direct band gap of 0.75 eV at zero tension (Figure 11a), which becomes indirect with a reduced gap of 0.2 eV when ε = 4% (Figure 11c). Within the tension range of 4% < ε < 10%, VS_2_-H monolayer is a semiconductor with anti-ferromagnetic ground state because the exchange energy is negative (Figures 10a, 11d & 11e). The anti-ferromagnetic VS_2_-H monolayer is metallic when 10% ≤ ε ≤ 16% (Figures 11f ~ i). When ε > 16%, VS_2_-H monolayer is ferromagnetic and half-metallic (Figures 10a & 11j). The evolutions of magnetism and conductivity of VSe_2_-H monolayer with tension (Figures 10b & 12) are similar to those of VS_2_-H monolayer (Figures 10a & 11). VS_2_-H monolayer is semiconducting and no-magnetic when ε ≤ 2%, semiconducting and anti-ferromagnetic when 2% < ε < 6%, metallic and anti-ferromagnetic when 6% ≤ ε ≤ 12%, and ferromagnetic and half-metallic when ε > 12% (Figures 10b & 12). According to the evolutions of conducting characters of VS_2_-H and VSe_2_-H monolayers, their anti-ferromagnetism under low tension is contributed to super-exchange for narrow-band semiconductor or carrier-enhanced super-exchange for metal, and their ferromagnetism under high tension is dominated by carrier-mediated double exchange in half-metal. The strong covalent bond between V and S/Se atoms in VS_2_-H and VSe_2_-H monolayers under zero or lower tension results in large band gap and less charge redistribution, contributing to their non-magnetic and semiconducting characters (Figures 10, 11a–c, & 12a–b). Comparing the exchange energies of hydrogenated vanadium dichalcogenides (VX_2_-H) monolayers (Figures 2&10), we see that the required tension at the turning point (magnetic or conducting switching) decrease with X in V-X bond changing as S → Se → Te because of its covalent bond weakening in the same trend and enhancement of charge redistribution.

## Conclusions

We present a first-principles study on the magnetic and electronic evolutions of hydrogenated vanadium dichalcogenides (VX_2_-H) monolayers under tension. Our calculations show that VTe_2_-H monolayer switches from anti-ferromagnetism to ferromagnetism via paramagnetic turning point accompanying with electronic evolution from semiconductor to metal, further to half-metal as tension increases. The anti-ferromagnetism of VTe_2_-H monolayer under low tension is contributed to the super-exchange in narrow-band semiconductor or mobile-carrier enhanced super-exchange in metal. The carrier-mediated double exchange in half-metal is attributed to the origin of ferromagnetism observed in VTe_2_-H monolayer under high tension. VS_2_-H and VSe_2_-H monolayers under lower tension are non-magnetic and semiconducting because the strong covalent bonds result in less charge redistribution. After tension exceeding a certain value, their magnetic and electronic evolutions under tension are similar to those of VTe_2_-H monolayer because of the charge redistribution and carrier-mediated interaction. We show that strain engineering, as a simple method, can efficiently control the magnetic and electronic properties of VX_2_-H monolayers. These monolayers with controllable functions can be applicable in mechanical sensors, functional nanodevices, and spintronics.

## Methods

The first-principles calculations are carried out to investigate the electronic and magnetic properties of vanadium dichalcogenide monolayer under tension. The calculations are based on the density functional theory (DFT)^36^ and the Perdew-Burke-Eznerhof generalized gradient approximation (PBE-GGA)^37^. The projector augmented wave (PAW) scheme^38^,^39^ as incorporated in the Vienna ab initio simulation package (VASP)^40^ is used in the study. The Monkhorst and Pack scheme of k point sampling is used for integration over the first Brillouin zone^41^. A 15 × 15 × 1 grid for k-point sampling for geometry optimization of unit cells, and an energy cut-off of 450 eV are consistently used in our calculations. Sufficiently large supercells are used so that the monolayers in neighboring cells in the vertical direction are separated by a vacuum region of at least 20 Å. A 2 × 2 × 1 cell is used to study the spin alignments. Spin-polarized calculations are employed. Full structural optimization is carried out for all the systems with tension before investigating their physical properties. Good convergence is obtained with these parameters and the total energy was converged to 2.0 × 10^−5^ eV/atom.

## Author Contributions

H.P. conceived the idea, performed the calculations, and wrote the paper.
